# The Effect of Calcineurin Inhibitors on MMPs Activity in Heart and Their Side Effects—A Review of Literature

**DOI:** 10.3390/ijms241210291

**Published:** 2023-06-18

**Authors:** Anna Surówka, Piotr Prowans, Michał Żołnierczuk, Marek Miśkiewicz, Tomasz Wawrowski, Marika Skodda, Marta Markowska, Karolina Kędzierska-Kapuza

**Affiliations:** 1 Department of Plastic, Endocrine and General Surgery, Pomeranian Medical University, 72-010 Szczecin, Poland; 2 Department of Plastic and Reconstructive Surgery, 109 Military Hospital, 70-111 Szczecin, Poland; 3 Department of Gastroenterological Surgery and Transplantology, National Medical Institute of the Ministry of Interior Affairs and Administration, 02-507 Warsaw, Poland; 4 Department of Gastroenterological Surgery and Transplantology, Centre of Postgraduate, Medical Education in Warsaw, 02-507 Warsaw, Poland

**Keywords:** metalloproteinases, calcineurin inhibitors, cyclosporine A, tacrolimus

## Abstract

This review focuses on the role of metalloproteinases in the pathogenesis of myocardial injury in various disease entities. It reveals how the expression and serum levels of metalloproteinases and their inhibitors change in many disease states. At the same time, the study offers a review of the impact of immunosuppressive treatment on this relationship. Modern immunosuppressive treatment is based mainly on the use of calcineurin inhibitors, including cyclosporine A and tacrolimus. The use of these drugs may carry a number of side effects, specifically to the cardiovascular system. The scale and degree of long-term influence on the organism remains unclear, but a significant risk of complications for transplant recipients who take immunosuppressive drugs as part of their daily treatment is to be expected. Therefore, the knowledge on this subject should be expanded and the negative effects of post-transplant therapy minimized. Immunosuppressive therapy plays an important role in the expression and activation of tissue metalloproteinases and their specific inhibitors, which leads to many tissue changes. The presented study is a collection of research results on the effects of calcineurin inhibitors on the heart, with particular emphasis placed on the participation of MMP-2 and MMP-9. It is also an analysis of the effects of specific heart diseases on myocardial remodeling through inductive or inhibitory effects on matrix metalloproteinases and their inhibitors.

## 1. Introduction

The number of potential organ recipients is constantly increasing, consequently the use of immunosuppressive therapy is becoming more frequent [1]. Immunosuppressive therapy is essential for all patients receiving an organ transplant, and its main task is to suppress the recipient body’s immune response. It is the only effective method used in transplantation to prevent organ rejection and enable long-term graft survival [2]. Calcineurin inhibitors are among the immunosuppressive drugs commonly used to suppress the body’s inflammatory response after organ transplantation. The aim of their action is a protein with serine-threonine phosphatase activity, involved in gene transcription and the synthesis of many cytokines involved in the body’s immune reactions. The increase in calcineurin activity is a consequence of the stimulation of T lymphocyte receptors by foreign antigens. Tacrolimus, by binding to a specific immunophilin, inhibits the T-cell signaling pathway. Cyclosporine binds calcineurin to a protein called cyclophilin, thus preventing dephosphorylation of nuclear factors of activated T-cells. Inhibition of this protein leads to decreased transcription of genes for IL-2, IL-3, IL-4, IL-5, TNF-alpha, and other cytokines involved in the induction of the inflammatory response, interfering with the differentiation, proliferation, and activation of T lymphocytes [3,4,5]. The expected therapeutic effect of immunosuppressive drugs also carries serious side effects, such as toxicity identified in a number of internal organs, including the liver (hepatotoxicity) or the kidneys (nephrotoxicity) [6,7]. Moreover, calcineurin inhibitors also have negative effects on the cardiovascular system. They lead to myocardial hypertrophy and fibrosis, and can cause cardiac arrhythmias, hypertension, as well as atherosclerotic vascular remodeling and lipid disorders [8]. Although chronic kidney failure increases the risk of cardiovascular incidents, when compared against the entire population the recipients of this organ are still at a higher risk of diseases related to the cardiovascular system [9,10,11]. The high survival rate of patients with functioning kidney transplants is an undeniable success of this branch of medicine. Unfortunately, myocardial and vascular lesions become the main cause of death in post-transplant recovery. It should be noted that at the initial stage of treatment this group of patients already suffers from many factors that increase the risk of CVD, such as diabetes, hypertension, and dyslipidemia, and some are incorrigible smokers [12,13]. The adverse effects of immunosuppressive treatment can accelerate the development of cardiovascular disease, eventually leading to coronary artery disease, heart failure, pulmonary hypertension, atherosclerosis, or aortic aneurysms [6,7,12].

In earlier studies, the effects of chronic immunosuppressive treatment on morphological changes in the aorta in the course of an imbalance between metalloproteinases (MMP-2 and MMP-9) and their inhibitors (TIMP-1 and TIMP-2) were investigated [9,10]. Metalloproteinases play an important role in the transformation function of the extracellular matrix of many tissues and structures of the body, including the heart [14]. This article focuses on the role of MMPs in the pathogenesis of myocardial injury, while also reviewing the available studies on the impact of immunosuppressive treatment on this interdependence. The data gathered in the course of the study will provide a research angle in the future research and following publications.

## 2. Structure of the Heart Wall

The heart is an organ consisting of two atria and two ventricles, whose function is to supply blood to all cells of the human body [15]. The walls of both the atria and ventricles are made up of numerous layers of cells, the arrangement of which allows the organ to work most efficiently. The endocardium is the innermost layer of the heart. It is built of monolayer squamous epithelial cells, which are an extension of the epithelial continuity of the blood vessels connected to the heart. Beneath the endocardium there is a layer of loose connective tissue containing blood vessels, nerves, and bundles of Purkinje fibers [16,17]. Myocardium consists of numerous cardiomyocytes, forming a branched structure in a way that ensures the most efficient contraction of the heart [18]. The outer layer of the heart, pericardium, consists of two parts—the visceral pericardium (endocardium) and the parietal pericardium. This structure ensures that the two pericardial plaques slide properly during each contraction [19].

To understand the structure of the entire heart in more detail, it is also necessary to look at the extracellular matrix (ECM). It is a complex network of fibrillar and non-fibrillar molecules located in the extracellular space, which provides the cement for the cells of the organ. Much of the ECM is made up of several types of proteins with structural, enzymatic and signaling functions. Albumins, type I, type III and type IV collagen, glycosaminoglycans, proteoglycans, non-collagen glycoproteins and elastins are among the aforementioned types of proteins. The ECM also contains numerous types of chemokines, cytokines and proteases, including metalloproteinases [20]. Understanding the structure and function of the extracellular matrix plays a significant role in tracking the mechanisms of inflammation, tissue remodeling and fibrosis, but also in heart regeneration. Factors causing damage to cardiomyocytes also stimulate the development of local inflammation. Increased vascular permeability allows the influx of stimulants provoking the inflammatory response, also contributing to the increase in the expression and activity of metalloproteinases. Under the influence of these proteins, the extracellular matrix is remodeled, thus the heart tissue is reorganized [21].

## 3. Metalloproteinases

Matrix metalloproteinases (MMPs) are a series of proteins with specific proteolytic properties, whose activity has a huge impact on the structure of the extracellular matrix, thus affecting the structure of the entire tissue. MMPs act either directly by proteolysis of tissue elements or indirectly by binding to cell surface receptors or inflammatory mediators. The main purpose of these proteases is to maintain balance between the synthesis and degradation of the components of the extracellular matrix. They play an important role in normal embryogenesis, the development of blood vessels, bone remodeling, tissue healing processes, scar formation, and in many other processes. Thus, the imbalance may be provoked by an excessive increase in the expression and activity of MMPs. This leads to pathological changes in tissues, such as carcinogenesis [14,22,23].

The classification of metalloproteinases is based on structural affinity of each MMP with one of five groups: collagenases: gelatinases, matrilysins, membrane-type metalloproteinases, stromelysins and unclassified metalloproteinases. The division is based on the substrate specificity of individual MMPs and differences in the quaternary structure of the protein. Despite that fact, the general structure of endopeptidases and the mechanism of action are largely uniform for all proteins [24,25].

Most proteins contain three domains: the catalytic domain, the hemopexin domain and the prodomain. The catalytic domain has an active site, which gives the metalloproteinase its proteolytic function. The structure of the active site depends on the substrate affinity of the MMPs. The hemopexin domain is responsible for regulating the activity of endopeptidases. The combination of this domain with the inhibitors of metalloproteinases causes a decrease in protein activity. Both protein fragments are connected by a stabilizing chain of amino acids. The prodomain holds the enzyme in the form of an inactive protein—pro-MMP. Removal of the propeptide protecting the active site results in full activation of the metalloproteinase [26].

The inactive form of the enzyme is secreted by most tissue cells, such as muscle cells, endothelial cells, fibroblasts, cells of the immune system (macrophages, leukocytes) or even cancer cells. Synthesis of endopeptidases is stimulated by, among others: cytokines (IL-1, IL-6), growth factors (EGF, FGF, VEGF, PDGF, HGF), tumor necrosis factors (TNF-α, TNF-β), antigens (CD40), free radicals, plasmin, thrombin, urokinase, and nitric oxides. The expression and activation of metalloproteinases is regulated by gene transcription, secretion of inactive forms of the enzyme by cells, and zymogen activation itself. Full activation is achieved by exposing the active site [27,28].

Tissue metalloproteinase inhibitors (TIMPs) are responsible for inhibiting the proteolytic activity of enzymes. The regulation is carried out by combining TIMP with the MMP active site, preventing the substrate from binding (non-competitive inhibition). Breaking the connection results in the re-activation of metalloproteinases. Four homologous forms of metalloproteinase inhibitors are known: TIMP-1, TIMP-2, TIMP-3, and TIMP-4. All TIMPs are similar in structure, but differ in affinity to individual MMPs and expression dynamics. The activity of these proteins is also regulated by growth factors and cytokines [29,30].

As mentioned above, metalloproteinases are classified according to their substrate specificity. In the cardiovascular system, the gelatinases MMP-2 (gelatinase A) and MMP-9 (gelatinase B) are the most active. They are characterized by the ability to decompose such tissue proteins as collagen types III, IV, V, VII, IX, XI, proteoglycan, elastin, fibronectin or laminin. Owing to their activity, gelatinases provide a space that allows the migration and proliferation of cells. The specific inhibitors for MMP-2 and MMP-9 are TIMP-2 and TIMP-1, respectively [24,31].

Under physiological conditions, cells control the secretion of MMPs and their inhibitors at a constant level. This allows for a balance to exist between degradation and the synthesis of tissue elements. Under the influence of stress factors and in response to the ongoing inflammation, there is a significant increase in the expression of metalloproteinases. The activity of endopeptidases is further exacerbated by a continuous increase in pro-inflammatory cytokines and tumor necrosis factors. Activated metalloproteinases hydrolyze extracellular matrix proteins and stimulate smooth muscle cell migration and proliferation. Tissue integrity and homeostasis is maintained by the balance and dynamics with which the ECM interacts with cells. Disturbances in this process can lead to the development of pathological cardiac and vascular changes, such as aneurysms, chronic venous insufficiency, atherosclerosis, coronary artery disease, myocarditis, heart failure, endocarditis and cardiomyopathies [28,32,33,34,35,36,37,38,39,40,41,42,43,44,45,46,47,48,49,50,51,52,53].

## 4. Clinical Significance of Metalloproteinases and Metalloproteinase Inhibitors in Cardiac Diseases

In many cardiac diseases, changes in the activity and cellular expression of a number of metalloproteinases, including MMP-1, MMP-2, MMP-3, MMP-7, MMP-8, MMP-9, MMP-11 and MMP-14, as well as their inhibitors, TIMP-1 and TIMP-2, are observed. They are involved in the development of apoptosis, inflammatory processes, remodeling and damage. In addition, they are an important element in maintaining the structural integrity of the heart [35]. A clinical understanding of the function of matrix metalloproteinases allows for a more thorough comprehension of the processes involved in myocardial damage and regeneration in specific cardiac disease entities.

Many authors point to the involvement of metalloproteinases in ischemic heart disease. It has been shown that in myocardial damage and subsequent reperfusion the ECM remodeling occurs, in which MMP-2, MMP-9 [36] and MMP-1 and TIMP-1 play a role [39]. The authors point out that the importance of those enzymes in muscle remodeling after myocardial infarction is not yet fully understood. Their importance may be clinically relevant for predicting impaired myocardial mechanics, and may also indicate the process of cardiac remodeling. In turn, some suggest that an imbalance in the concentrations of metalloproteinases, particularly MMP-2 and MMP-9, occurs in the process of atherosclerosis development in the coronary vessels [37,38,40]. This phenomenon leads to destabilization of the atherosclerotic plaque and promotes the induction of an acute coronary incident. For this reason, some reports treat the imbalance of metalloproteinase activity as one of the cardiovascular risk factors [40].

In viral myocarditis, inflammatory cell infiltration contributes significantly to the release of inflammatory cytokines and metalloproteinases. These are involved not only in cardiac remodeling after inflammation, but also in regulating the acute phase of the inflammatory process in the heart [42,43]. In addition, it has been detected that elevated activity of metalloproteinases, most notably MMP-2, can lead to abnormal vascular remodeling through increased migration of vascular wall smooth muscle cells into the intima, and can also result in increased fibrosis and reduced elastin content. This phenomenon correlates strongly negatively with left ventricular ejection fraction in patients with myocarditis [41].

There are also studies whose authors investigated the relationship between atrial fibrillation and the expression of metalloproteinases in the atria of the heart. They noted that MMP-9 plays an essential role in pathological structural remodeling of the atria. This process promotes the occurrence and persistence of AF symptoms [44,45,46,47].

Researchers have also conducted studies among post-heart-failure patients. One study noted that increased levels of MMP-2 in heart failure correlate with an increased risk of death and disease-related deterioration and hospitalization [48]. In contrast, another research work showed that MMP-1 levels are decreased in heart failure, while TIMP-1 levels are increased, resulting in increased interstitial fibrosis in the ECM through increased deposition of type I and III collagen. This process increases myocardial stiffness and causes impaired contraction, which can cause further progression of the disease [49].

In contrast, an increase in circulating serum MMP-9 has been observed in infective endocarditis. The inflammatory process may result in the release of this enzyme from infected tissues or by the circulating macrophages and neutrophils involved in the inflammatory response. Overexpression of MMP-9 is associated with an increased risk of embolism. This fact is explained by an increase in the fragility of the infected tissue and bacterial vegetation, increasing the risk of embolism [50].

Metalloproteinases also play a role in disease processes associated with cardiomyopathy. An imbalance in the expression of MMPs in the heart is associated with pathological cardiac remodeling. Increased activity of MMPs (primarily MMP-2 and MMP-9) and decreased activity of TIMPs leads to under-regulation of local MMPs, and thus to cardiac remodeling and reduced cardiac contractility. This can lead to end-stage heart failure [51,52,53].

Long-term use of immunosuppressive therapy increases the morbidity and mortality due to cardiovascular diseases in all recipients, and also becomes the main cause of death in patients after kidney and heart transplantation. Treatment is associated with negative effects on the cardiovascular profile, such as hypertension, dyslipidemia and hyperglycemia. These are important risk factors for coronary heart disease (CAD) [54,55]. Morphological changes in the myocardium, including fibrosis and remodeling of the extracellular matrix, may be an expression of abnormal activity of metalloproteinases and appear as an independent risk factor for heart disease [56].

The changes in the levels and activities of metalloproteinases and their inhibitors in the above-mentioned heart diseases are summarized in the table below (Table 1).

## 5. Effects of Immunosuppressive Drugs on Cardiac MMPs Activity

Calcineurin is a cellular protein with serine/threonine phosphatase activity and signal transducer function in the pathway leading to T-lymphocyte activation [57]. The enzyme is composed of a catalytic subunit, calcineurin A, which binds calmodulin, and a regulatory subunit, calcineurin B, which binds calcium. Antigen binding to the T cell receptor leads to an increase in intracellular calcium. Calcium ions combine with calmodulin and calcineurin B to produce a calmodulin-calcineurin-Ca^2+^ complex [3]. The phosphatase thus activated influences the expression of relevant genes through the dephosphorylation of the nuclear factor of activated T cells (NFATc) [4]. De-phosphorylated forms of the enzymes move from the cytoplasm to the cell nucleus, where they contribute to increased transcription of genes for IL-2, IL-3, IL-4 and tumor necrosis factor (TNF-α), among others. The produced cytokines stimulate leukocyte proliferation and differentiation. Calcineurin inhibitors affect the pathway described above [58,59]. The immunosuppressive effect of cyclosporine is based on the binding of calcineurin to cyclophilin. This prevents the dephosphorylation of NFATc, thereby activating the transcription of genes for IL-2, IL-3, and IL-5, among others. Interleukin-2 is a key factor in the activation of T lymphocytes, interferon gamma and granulocyte and macrophage colony-stimulating factor. Inhibition at this level prevents T-cell activation, but also silences the general, humoral and cellular immune response. In addition to its immunomodulatory properties, CsA also has many side effects. The drug contributes to acute and chronic renal failure and hypertension. These complications can result from vascular endothelial damage [60,61,62].

The structure of the tacrolimus molecule is different from that of CsA the immunosuppressive effect being stronger than in cyclosporine [63]. Tacrolimus forms complexes with immunophilins defined as FK506-binding proteins (FKBP), and the combination stops the dephosphorylation of transcription factors. Cytokine expression and the cascade that stimulates T-cell proliferation and activity are not triggered. The drug does not affect phagocytic cells. Tacrolimus may affect the development of hypertension, angina pectoris and cardiomyopathy in children. Due to their strong inhibitory effect on the body’s immune response and their high efficacy in preventing rejection of the transplanted organ, calcineurin inhibitors are standardly used in immunosuppressive treatment regimens [62,64,65].

A study on the hearts of rats was conducted by a group of Swiss scientists in 2009. The rats were divided into two study groups—one group received an allograft heart transplant and was subsequently given a dose of cyclosporine A (CsA). The other group received an allograft without subsequent CsA therapy in order to represent in the study the group manifesting acute graft rejection (AR). The control group consisted of hearts from healthy rats. After 5 days, hearts were harvested from both groups and the expression and activity levels of individual metalloproteinases and their inhibitors were examined immunohistochemically. The outcomes were compared with those of the control group.

Heart samples from untreated animals demonstrated an increase in mRNA levels for a number of metalloproteinases, including MMP-2, MMP-9 and TIMP-1, and a reduction of mRNA levels for TIMP-2. In comparison with the group not receiving the calcineurin inhibitor, CsA lowered mRNA levels for only one metalloprotease inhibitor—TIMP-1.

Cyclosporin A treatment also led to a decrease in MMP-2 mRNA expression, but the TNF-α levels in this group were similar to the control group, while the acute graft rejection group showed a significant increase in MMP-2 and TNF. This phenomenon indicates an ongoing inflammatory reaction that is a defensive response to tissue damage in rats undergoing cardiac allograft. Graft rejection significantly increases the expression of extracellular matrix proteins (collagen, laminin and fibronectin), which are the leading substrates for MMP-2 and MMP-9. The use of the immunosuppressive drug fosters this effect, which is a crucial observation in that particular study. A very important aspect of these studies was to analyze not only the expression of MMPs, but also to demonstrate the presence of latent forms of proteins. The expression of the inactive forms of proteases—pro-MMP-2 and pro-MMP-9 increased in the AR group. A rise in the pro-MMP-9 activity in zymography was also noted. Cyclosporine exposure had literally the opposite effect, that is, a decrease in the level of latent forms and in the activity of pro-MMP-9. It should be emphasized that the expression of active gelatinases was unchanged in all study groups. The researchers noted that due to the fact that the study was conducted shortly post-transplant, active forms of metalloproteinases might not have been displayed. It cannot be ruled out that CsA may have a suppressive effect on MMP-9 activity independently of gene expression.

Based on the described research work, it can be stated that the process of acute graft rejection demonstrates a positive correlation with increased gene expression of MMPs, TIMPs and ECM elements. The implementation of short-term CsA therapy reduces the process of acute graft rejection mainly by reducing the influx of mononuclear cells. The benefits obtained may be related to the negative pro-fibrotic effect of the drug manifested by increased expression of TIMPs and extracellular matrix constituents [66].

The effects of cyclosporine on the hearts of rats was also examined by Italian scientists. The test group was injected subcutaneously with a dose of CsA for twenty-one days. The control group consisted of rats receiving a subcutaneous dose of castor oil. The experiment ended with the euthanasia of the animals. Heart samples were harvested and examined.

The obtained results showed negative effects of the calcineurin inhibitor on the myocardium. Advanced degenerative changes in muscle fibers and even disorganization of the structure were observed after using the drug in animals. Accumulations of connective tissue and an increased amount of collagen fibers were also noted. Most importantly, the expression of gelatinase A and vascular endothelial growth factor (VEGF) were at a very high level.

In their conclusions to the study, the authors emphasized that the increase in the level of vascular endothelial growth factors has in this case a beneficial effect on the preservation of heart function. Factors VEGF is part of a defense mechanism designed to protect cells from ischemic and subsequent damage. It may also stimulate the expression of MMP-2 as a prevention of cardiac tissue fibrosis. Counteracting to reduce cyclosporine-induced myocardial fibrosis occurs by increasing the activity of proteolytic enzymes. To sum up, that specific study shows that the increase in VEGF and MMP-2 levels acts as a compensatory mechanism in response to the toxic and profibrotic effects of CsA on the heart [56].

In another trial, the researchers reported on the dynamics of changes in serum MMP and TIMP levels in heart transplant patients who received calcineurin inhibitors as their main immunosuppressive agent. The study included heart transplant recipients, who represented two treatment groups. The first one received a cyclosporine-based regimen, mycophenolate mofetil, and a corticosteroid. The other group received tacrolimus instead of cyclosporine. The use of both calcineurin inhibitors resulted in an increase in the concentration of MMP-1, MMP-8, MMP-9 and TIMP-1 in the serum during the first three weeks of treatment. After this period, protein concentrations gradually decreased. There was no correlation between the concentration of proteolytic enzymes or their inhibitors and the graft ischemia time.

Unfortunately, specific data on the individual treatment regimens included in the study were not provided. Nevertheless, the outcomes described confirm the extent and impact of metalloproteinases on the process of cardiac remodeling and regeneration after a period of ischemia. The authors also point to the possible involvement of MMPs in the process of graft rejection. In fact, increased serum levels of MMP-1 were correlated with episodes of graft rejection. Decreased serum MMPs levels may also result from immunosuppressive therapy. The use of calcineurin inhibitors, as in the work of Berthier CC et al., can initiate fibrotic processes [67].

In a subsequent study, myocardial infarction (MI) was provoked in mini-pigs by ligation of the left anterior descending artery. After an initiated myocardial infarction, a group of mini-pigs (MI-Tac) received an intravenous dose of tacrolimus. Another group of pigs (MI only) received saline. The control group (NC) consisted of heart tissue from six healthy animals. The pigs were put down and the hearts were sourced for testing.

The expression of mRNA for MMP-9, NF-κB, TNF-α and PAI-1 in the heart was significantly higher in untreated pigs after MI compared to the control group. However, the aforementioned markers were significantly lower in the group treated with tacrolimus after MI. That specific study was able to demonstrate the effect of an immunosuppressive drug on the expression of inflammatory mediators. Thus, tacrolimus inhibits the activation of the inflammatory response after an episode of acute myocardial ischemia. Ongoing inflammation after an ischemic episode also increases the area of muscle damage. Tacrolimus visibly reduced degenerative progress in the heart, which contributed to the preservation of left ventricular function. This information is essential for the management of acute MI without reperfusion therapy. The authors also noted that the calcineurin inhibitor group had significantly less tissue fibrosis compared to the untreated group. The occurrence of cell apoptosis was also less severe in this group.

There was a marked effect of tacrolimus in reducing the levels of inflammation-inducing molecules (PAI-1, NF-κB and TNF-alpha), as well as its antifibrotic effect. Limited number of substrates for MMP-9, may be responsible for the lack of a compensatory increase in mRNA expression for this metalloproteinase [68].

## 6. Commentary

Analyzing the above results, it should be noted that there is a potential correlation between MMP expression in heart ECM and administration of immunosuppressive drugs and it calls for further studies in clinical trials. There is evidence that the administration of immunosuppressive drugs directly affects the expression of many types of MMPs in the heart (Table 2). Information about aforementioned immunosuppressive drugs is presented in separate table (Table 3).

There are no studies on the effect of other immunosuppressive drugs, such as mTOR-pathway inhibitors or mycophenolate mofetil, on balance between matrix metalloproteinases (MMP-2 and MMP-9) and their inhibitors in heart. This review is an introduction to further research on the influence of immunosuppressive treatment regimens on morphological changes in the heart, with particular consideration of the balance between matrix metalloproteinases and their inhibitors.

## 7. Conclusions

Calcineurin inhibitors as immunosuppressive drugs widely used in transplantation enable longer survival of transplanted organs. In contrast, these drugs affect the imbalance between ECM components, including metalloproteinases, contributing to adverse effects. Unfortunately, the small number of studies on the effects of these drugs on the heart prevents a thorough understanding of the pathophysiological processes occurring in the extracellular matrix after immunosuppressive treatment.

## Figures and Tables

**Table 1:** Individual MMP and TIMP activity and expression in heart diseases.

Disease Entity	Increase in Enzyme	Decrease in Enzyme	Reference
Coronary artery disease	MMP-2	---	[36,37,38,39,40]
	MMP-9		
	MMP-1		
	TIMP-1		
Myocarditis	MMP-2	---	[41,42,43]
	MMP-3		
	MMP-8		
	MMP-9		
	MMP-12		
	TIMP-2		
	TIMP-4		
Atrial fibrillation	MMP-9	MMP-1	[44,45,46,47]
	TIMP-1		
Heart failure	MMP-2	MMP-1	[48,49]
	MMP-9		
	TIMP-1		
Endocarditis	MMP-9	---	[50]
Cardiomyopathies	MMP-2	MMP-1	[51,52,53]
	MMP-7		
	MMP-8		
	MMP-9		
	MMP-14		

**Table 2:** Effects of immunosuppressive drugs on heart’s MMPs.

Drug Name	Metalloproteinases Activities	Examined Material	Comments	References	Country
CALCINEURIN INHIBITORS					
CYCLOSPORINE A	Decreased mRNA levels of MMP-7, TIMP-1 and TIMP-3. Increased mRNA levels for MMP-11, MMP-16 and MMP-23. Decreased activity of MMP-9 and MMP-14 production	Rat heart allograft cells.	Drug improved histology of graft rejection and showed potential fibrosis-promoting effects.	[66]	Switzerland
	Increased expression of MMP-2 and VEGF. No effect on MMP-1 and MMP-9.	Rat cardiomyocytes.	Hearts of CsA-treated rats showed degenerative changes, muscle fibers became disorganized, connective tissue was present in greater amounts compared to the control sample.	[56]	Italy
	Increased serum levels of MMP-1, MMP-8, MMP-9 and TIMP-1 after 2–3 weeks. After that time— gradual decrease.	Serum samples and endomyocardial biopsies of heart transplant patients.	Combination therapy with mycophenolate mofetil and glucocorticosteroids.	[67]	Austria
TACROLIMUS	Inhibition of MMP-9 expression.	Pig hearts.	Drug showed reduced MMP-9 expression after myocardial infarction compared to a trial without tacrolimus.	[68]	Taiwan
	Increased serum levels of MMP-1, MMP-8, MMP-9 and TIMP-1 after 2–3 weeks. After that time— gradual decrease.	Serum samples and endomyocardial biopsies of heart transplant patients.	Combination therapy with mycophenolate mofetil and glucocorticosteroids.	[67]	Austria

**Table 3:** Information on immunosuppressive drugs used in the above studies (test groups).

Study	Drug Details	Dose and Time of Administration	Form of Administration of the Drug	Quality Control
Berthier et al. [66]	Cyclosporine A (Neoral, nd *)	7.5 mg/kg/day for 5 days after transplantation	No information	No information
Bianchi et al. [56]	Cyclosporine A (Sandimmun, Sandoz, nd)	15 mg/kg/day for 21 days	Subcutaneously	No information
Aharinejad et al. [67]	Methylprednisolone (nd)	500 mg intra-operatively and 125 mg at 8, 16 and 24 h post-transplant	Intravenously	No information
	Polyclonal anti-thymocyte globulin (Thymoglobuline, Cambridge, MA, USA)	2 mg/kg for 3–7 days	Intravenously	No information
	Cyclosporine A (Sandimmun Neoral, Novartis, Basel, Switzerland)	Individual doses unknown—target serum levels 200–250 ng/mL, treatment started 3–7 days after transplantation	Orally	No information
	Tacrolimus (Prograf, Astellas Pharma, Deerfield, IL, USA)	Individual doses unknown—target serum levels 12–15 ng/mL, treatment started 3–7 days after transplantation	Orally	No information
	Mycophenolate mophetil (Cell Cept, Hofmann-La Roche, Grenzach-Wyhlen, Germany)	1.5 g–3 g a day	Intravenously on the first postoperative day, then orally	No information
	Prednisolone (nd)	5–15 mg at alternating daily doses	Orally	No information
Chua et al. [68]	Tacrolimus (nd)	0.5 mg in 2.5 mL saline (once)	Intra-coronary administration	No information

* nd—no data.
